# 

**Published:** 1838-04

**Authors:** 


### BOOKS RECEIVED FOR REVIEW.

ENGLISH.

1. Researches into the Physical History of Mankind. By J. C. Prichard, M.D. F.R.S. M.R.I.A. &c. Third Edition, Vol. II.—London, 1837. 8vo. pp. 373. 15s.

2. Outlines of the Principal Diseases of Females. By Fleetwood Churchill, M.D., Lecturer on Midwifery, &c. Dublin.—Dublin, 1838. 8vo. pp. 402. 10s. 6d.

3. A concise Treatise on Operative Surgery, describing the method adopted by the English, Continental, and American Surgeons; selected for the use of junior Practitioners and Students. Illustrated by 12 Plates. By W. P. Cocks, Surgeon.—London, 1837. 8vo. pp. 375. 14s.

4. A Letter to Lord John Russell, M.P., Secretary of State for the Home Department, on the evil Policy of Quarantine and Restrictive Police employed against the Asiatic Cholera, &c. By Joseph Ayre, M.D.—London and Hull, 1837. 8vo. pp. 39.

5. Transactions of the Medical and Physical Society of Calcutta. Vol. VIII. Part I.—Calcutta, 1836. 8vo. pp. lxxix. 171.

6. The Quarterly Journal of the Calcutta Medical and Physical Society. Edited by H. H. Goodeve, M.D., and W. B. O'Shaughnessy, M.D. Nos. I. and II. January, April, 1837.

7. An Essay on the Antiquity of Hindoo Medicine; including an introductory Lecture to the Course of Materia Medica and Therapeutics, delivered at King's College. By J. F. Royle, M.D. F.R. & L.S., Sec. G.S., &c. Professor of Materia Medica and Therapeutics, King's College, London.—London, 1837. 8vo. pp. 190. 6s. 6d.

8. The Works of John Hunter. Edited

LIBRARY OF THE

Orleans Parish Medical Society by J. F. Palmer. Vol. IV. Containing Observations on certain parts of the Animal Economy; with Notes, by Richard Owen, F.R.S.—London, 1837. 8vo. pp. 506. 17s. 6d.

9. Ophthalmia. The various Inflammations of the Conjunctiva or Mucous Membrane of the Eye. By J. Slade, M.D. &c.—London, 1838. 8vo. pp. 120.

10. Elements of Chemistry. By the late E. Turner, M.D. Sixth Edition, Part II.—1837. 8vo. 5s.

11. Elements of Anatomy. By Jones Quain, M.D. Fourth Edition, Part II.—London, 1838. 8vo. 5s.

12. Report of the Proceedings under a Brieve of Idiotcy, Duncan against Yoolow, tried at Cupar-Angus, 28-30 Jan. 1837. By L. Colquhoun, Esq., Advocate.—Edinburgh, 1837. 8vo. pp. 135.

13. On the Statistics of English Lunatic Asylums, and the Reform of their public Management. By William Farr.—London, 1835. 8vo. pp. 46. 2s.

14. The Evils of Quarantine Laws, and Non-existence of Pestilential Contagion; the Privy Council and College of Physicians; the Means of Prevention and Method of cure of the Cholera Morbus, and the atrocities of the Cholera Panic. By Capt. White, late H. E. C. Service.—London, 1837. 8vo. pp. 176. 8s.

15. Medicine and Surgery one inductive Science; being an attempt to improve its Study and Practice, &c. By George Macilwain, Surgeon to the Finsbury Dispensary, &c.—London, 1838. 8vo. pp. 551. 12s.

16. Appendix to the eighth Edition of The Pharmacologia; with some Remarks on various Criticisms upon the London Pharmacopœia of 1836. By J. A. Paris, M.D. F.R.S. &c.—London, 1838. 8vo. pp. 31. 2s. 6d.

17. The Cyclopædia of Anatomy and Physiology. Edited by R. B. Todd, M.D. Part XIII. (Gasteropoda - Gland.)—London, February 1838. 5s.

18. Illustrations of the Comparative Anatomy of the Nervous System. By Joseph Swan. Part III.—London, 1838. 4to. 7s.

19. The Student's Companion to Apothecaries' Hall; or, the London Pharmacopœia of 1836 in Question and Answer. By E. Oliver, M. R. C. S. L.—London, 1837. 18mo.

20. The Stomach in its Morbid States; being a practical Inquiry into the Nature and Treatment of Diseases of that Organ, &c. By Langston Parker, M.R.C.S.—London, 1838. 8vo. pp. 303. 10s. 6d.

21. Observations on the Influence of Climate on Health and Mortality. A Prize Thesis. By A. S. Thomson, M.D.—Edinburgh, 1837. 8vo. pp. 102.

22. On Warming and Ventilating; with Directions for making and using the Thermometer Stove, or self-regulating Fire, and other new apparatus. By Neill Arnott, M.D. Physician Extraordinary to the Queen.—London, 1838. 8vo. pp. 138. 5s.

23. A short Treatise on the External Characters, Nature, and Treatment of the different forms of Porrigo. By Walker Dick, M.D. Glasgow, 1838. 8vo. pp. 58.

24. Elements of Anatomy. By Jones Quain, M.D.—London, 1838. 8vo. pp. 910, with Plates and Woodcuts. 22s.

25. Statistical and Pathological Report of the Royal Infirmary of Edinburgh for the Years 1833-7. By John Hare, M.D. (From the Edinburgh Med. and Surgical Journal.)—Edinburgh, 1838. 8vo. pp. 31.

26. A Treatise on Ruptures. By W. Lawrence, F.R.S., Surgeon Extraordinary to the Queen, Surgeon to St. Bartholomew's Hospital, &c. Fifth Edition, revised, corrected, and considerably enlarged.—London, 1838. 8vo. pp. 632. 16s.

27. Facts connected with the Medical Relief of the Poor in the Bridgwater Union.—London, 1838. 8vo. pp. 22.

28. Ophthalmic Memoranda respecting those Diseases of the Eye which are most frequently met with in Practice. By John Foote, F.R.C.S.—London, 48mo. pp. 90. 1s.

29. Notes on the Medical History and Statistics of the British Legion of Spain; comprising the Results of Gun-shot Wounds, in relation to important Questions in Surgery. By Rutherford Alcock, K.T.S. &c., Deputy Inspector General of Hospitals, &c.—London. 1838. 8vo. pp. 101. 5s.

30. An Experimental Inquiry into the Physiology of Cutaneous Absorption, and its Application to Therapeutics; being the Essay to which the Medical Faculty of the University of Edinburgh awarded a Gold Medal, at the Graduation on the 1st Aug. 1837. By W. H. Madden, M.D.—Edinb. 1838. 8vo. pp. 151. 5s.

31. A Treatise on some Nervous Disorders; being chiefly intended to illustrate those Varieties which simulate Structural Disease. By E. Lee, M.R.C.S. Second Edition, re-written and considerably enlarged; with an Appendix of Cases.—London, 1838. 8vo. pp. 176. 5s.

32. Reports and other Documents relating to the State Lunatic Hospital at Worcester, Massachusetts.—Boston (U. S.), 8vo. pp. 200.

33. The Nervous System of the Human Body; as explained in a Series of Papers read before the Royal Society of London: with an Appendix of Cases and Consultations. By Sir Charles Bell, K.G.H. F.R.SS.

L. and E., &c. — Edinburgh, 1836. 8vo. pp. 476, with numerous Plates. 24s.

34. Institutes of Surgery: arranged in the order of the Lectures delivered in the University of Edinburgh. By Sir Charles Bell, &c., Professor of Surgery. Vol. II. Edinburgh, 1838. 8vo. pp. 380. 7s. 6d.

35. The Substance of two Lectures upon the Arguments to be derived from the Physical Organization and Moral Constitution of Man, in Proof of his Religious Obligations. By William Brewer, M.D.— London, 1838. 8vo. pp. 54.

36. On Diseases of the Bladder. By Wm. Coulson, Surgeon.—London, 1838. 12mo. pp. 153. 5s.

37. The Hunterian Oration delivered in the Theatre of the Royal College of Surgeons in London, on the 14th February, 1838. By B. Travers, F.R.S., Surgeon Extraordinary to the Queen, &c.—London, 1838. Royal 8vo. pp. 42. 2s. 6d.

38. A Treatise on the Nature, Symptoms, Causes, and Treatment of Insanity; with Practical Observations on Lunatic Asylums, &c. By Sir W. C. Ellis, M.D., Resident Medical Superintendent of the Hanwell Lunatic Asylum.—London, 1838. 8vo. pp. 344. 10s.

FOREIGN.

1. Handbuch der Menschlichen Anatomie. Verfasst von C. F. T. Krause, M.D., Professor der Anatomie zu Hannover. Ersten Bande, I. u. II. Abtheilung. — Hannover, 1833–1836. 8vo. pp. 632.

2. Beiträge zur Aufhellung der Verbindung der menschligen Frucht mit dem Fruchthälter und der Ernähurung derselben. Von F. A. Ritgen, Professor der Wundarzneikunde, &c.— Leipzig und Stuttgart, 1835. Fol. pp. 78; mit drei Tafeln.

3. Der Mensch nach verschiedenen Seiten seiner Natur, oder Anthropologie für das gebildete Publikum. Von K.F.Burdach, Professor zu Königsberg, &c.—Stuttgart, 1837. 8vo. pp. 787; mit drei Kupfertafeln.

4. Einleitung zu den Affekten als Krankheitsursachen und Heilmittel, &c. Von Dr. C. G. Helbig, prakt. Arzte zu Dresden. —Leipzig, 1836. 8vo. pp. 100.

5. Grundzüge einer Künftigen speciellen Homöopathischen Therapie, &c. Von Dr. E. F. Rückert.—Leipzig, 1837. 8vo. pp. 531.

6. Vergleichende Idealpathologie. Ein Versuch die Krankheiten als Rückfälle der Idee des Lebens darzustellen. Von Dr. K. R. Hoffmann.—Stuttgart, 1834. 8vo. pp. 687.

7. Primæ Lineæ Pathologiæ humorum qui in Corpore Humano circulantur, ad præsentem Physiologiæ ac Pathologiæ statum constructæ. Scripsit C. H. Roesch, M.D. &c.—Stuttgardiæ, 1837. 8vo. pp. 119.

8. Untersuchungen aus dem Gebiete der Heilwissenschaft. Von Dr. C. Roesch, &c. Erster Theil.—Stuttgart, 1837. 8vo.

9. Prosp. Alpini Opera. Denuo Edidit. J. B. Friedreich.—Nordlingiæ, 1829. 4 Vol. 8vo.

10. Das Blutfieber, vorzüglich in seiner Verbindung mit einigen Krankheiten des Darmkanals. Pathogenisch und therapeutisch erläutert von F. Kehner.— Mainz, 1837. 8vo. pp. 128.

11. Ernesti de Grossi, M.D., Prof. &c. Opera Medica Posthuma. Curantibus discipulis S. Fischer et F. Pruner, M.D. — Stuttgardiæ, 1831. 3 Vol. 8vo.

12. De Artis Medicæ in Græcia statu hodierno. Scripsit C. G. Thraemer, M.D. e Medicis primoribus Classis Russicæ.— Dorpati Livonorum, 1836. 8vo. pp. 123.

13. Skizze ueber Algier in medicinischer Rücksicht. Vom Dr. A. von Schönberg, Königl. Danischen Archiater.—Kopenhagen, 1837. 8vo. pp. 106.

14. Questiones Emmenelogicas scripsit F. F. Krieg, M.D. — Lipsiæ, 1837. 4to. pp. 30.

15. Traité théorique et pratique de la Dérivation, contre les Affections les plus communes. Par L. F. Gendron, M.D. &c. —Paris, 1837. 8vo. pp. 331.

16. Cornelii Pruys Van der Hoeven, Initia Disciplinæ pathologicæ auditorum in usum edita.— Lugd. Batav. 1834. 8vo. pp. 364.

17. Institutio de Morbis acutis, sive Doctrina Inflammationis et Febrium. Auctore G. C. B. Suringar, M.D. —Amstelodami, 1836. 8vo. pp. 220.

18. Epitome Therapiæ generalis; in usum Discipulorum. Scripsit G. C. B. Suringar, M.D. &c.—Amstel. 1834. 8vo. pp. 150.

19. Isagoge in Doctrinam Morborum chronicorum. Auctore G. C. B. Suringar, M.D. Pars I.—Amstel. 1837. 8vo. pp. 210.

20. Aug. Arnoldi Sebastian, M.D., Medicinæ in Academia Groningana professoris ordinarii Physiologia Generalis.-Groningæ, 1835. 8vo. pp. 301.

21. Mémoire sur les Causes générales des Syphilides, &c. Par C. F. Martins, M.D. &c.—Paris, 1838. 8vo. pp. 110.

22. Ueber radicale Heilung reponibler Brüche. Von Dr. Ph. Finck. Mit ii. Kupfert.—Freiburg, 1837. 8vo. pp. 48.

23. Grundriss der speciellen Semiotik nach den Quellen bearbeitet. Von Dr. H. E. Suckow.—Jena, 1838. 4to. pp. 296.

24. Cercaria's Reise durch den Microcosmus oder humoristischer Ausflug in das Gebiet der Anatomie, Physiologie, und Medizin. Herausgegeben von Menapius. —Crefeld, 1836. 8vo. pp. 160.

### INDEX TO VOL. V.

OF THE

### BRITISH AND FOREIGN MEDICAL REVIEW.

PAGE

Aconite, its use in headach . 275
Acupuncture in hydrocele and ascites 279
Affusion of cold water in wounds . 574
Ahrensen, Dr. on the Endermic Method . . . . 343
Air in the veins, cause of death . 420
Albers, his observations in Pathology 187
Alison, Dr. on the Reflex Function . 533
America, mode of living in . . 409
Ammon, Dr. von, on Tenotomy . 550
Amputation, spontaneous, in utero . 614
Anatomy, Dr. Quain's Elements of . 216
Andral, his notes on Laennec . 423
Aneurism treated by ice . . 568
Anus, diseases of the . . 484
Antoninus, plague in the time of . 193
Aorta, on the valves of . . 272
Aortitis, a cause of angina pectoris . 273
Appointments, royal, in Scotland . 626
Arnold, MM. on the laws of the living body . . . 75, 80
Arnott, Dr. on Warming and Ventilating . . . . 546
Arntzenius, Dr. on Suicide . 368
Arteries, on the obliteration of . 564
Articulation, spino-occipital, disease of . . . . 278
Ascites, on acupuncture in . . 279
Asphyxia, Mr. Mayo on . 78, 98
 on artificial respiration in 277
Assurance Societies . . 311
Auscultation, Dr. Breventani on . 167
 Dr. Wolff on . . 215

Badham, Dr. on the sensibility of insects . . . . 545
Bafureira, a new medicine . . 563
Belladonna, endermic use of . 349
Beriberi, on the treatment of . 127
 Mr. Malcolmson's essay on 116
Berlin Lying-in Hospital, report of . 576
Billing, Dr. his Principles of Medicine . . . . 200
Birds, on the structure of the iris in 553
Bladder, case of ear-pick in . 253
 on soot in diseases of . 575
Blakiston, Dr. on the Influenza . 214

PAGE

Blane, Sir G. on the Spinal Cord . 529
Blood in cholera, experiments with . 223
 on animalculæ in . . 555
 mode of promoting the flow of 574
Blood-vessels, on diseases of the . 467
Bone, case of absorption of . 244
Books received for review . 315, 622
Bowditch, Dr. his translation of Louis 549
Boyer, Baron, his treatise on Syphilis, 229
Brain, Dr. Calori on the structure of 220
 severe injury of . . 236
 of the Negro . . 585
Brewster, Sir D. on Cataract . 602
Bronchi, foreign bodies in . 440
Bronchotomy in laryngitis . 430, 441
Bull, Dr. his Hints to Mothers . 215
Burning of the feet, an Indian disease, 131
Busch, Dr. his Berlin Midwifery Report . . . . 576
Buxton, bathing at . . 397

Cæcum, rupture of, in labour . 256
Cainca, a new remedy . . 562
Calomel, external use in ophthalmia 251
Capillary circulation, independence of, 337
Carus on the development of the ovum, 555
Carpenter, Dr. on sensation . 331
Carotid, aneurism of the . . 610
Casper, Dr. on Hanging . . 615
Cataract, Sir D. Brewster on . 602
 on the extraction of . 285
Chemistry, medico-legal, Gusserow on, 212
 Dr. Turner's . . 216
Chest, Dr. Stokes on diseases of . 423
 instrument for measuring . 425
Cholera, Dr. Versari on . . 169
 Dr. Bellingeri on . . 234
 in Venice . . 259
 in Naples . 260, 625
 treatment of, by lead and opium . . . 280
 probability of life in . 292
Circulation, Mr. Wardrop's views of the, 146
Club-foot, division of tendon in . 288
Clutterbuck, Dr. on Pyrexia . 185
Cocks, Mr. his Operative Surgery . 544
Cogswell, Dr. on Iodine . . 162

PAGE
Colles, Mr. on Syphilis . 1, 27
Constitution, hereditary, modified . 406
Contractility, its characteristics . 487
Corrigan, Dr. on Aortitis . . 273
Cotton, its use in erysipelas . 244
Cowan, Dr. on the stethoscope . 267
Cranium, deficient ossification of . 273
Croup, account of . . . 437
Cullen, Dr. on the Spinal Cord . 526
Cupping-glass, new, account of . 252
Curtis, Mr. on Health . 380, 408
Cutaneous diseases, on caustic in . 287
Cutlers, on diseases of . . 257

Davidson, Dr. his Glasgow Report . 607
Delirium tremens, treatment of . 242
Dr. Munk on . 600
Delivery, unconscious . . 255
Desruelles, M. on Syphilis . 1, 25
Diet, Drs. Paris and Robertson on . 380
Dieterich, on Mercurial Disease 2, 29
Dilatation, sudden, in stricture . 572
"Discoverers, the Rival" . . 621
Dislocation of the shoulder-joint . 569
Dissection in Paris, history of . 452
Diuretics, endermic use of . 350
Dropsy of the ducts and glands . 187
Duchatel on Hygiène . . 447
Dunglison, Dr. his Physiology 75, 113
Dysentery treated by sugar . 443

Ear, on diseases of the . . 216
Earle, Mr. obituary of . . 627
Education, physical, of women . 405
Edwards, Dr. M. his Elements of Zoology . . . . 204
Eggert, Dr. obituary of . . 314
Embalming, Tranchina on . . 221
Empyema, new mode of treating . 242
new treatment of . 573
Endermic method, dissertation on . 343
Enemata, mode of using . . 392
Epiphyses of bones, separation of . 250
Erysipelas, Dr. Williams on . 365
treatment of, by cotton . 244
Expectorants, endermic use of . 351
Experiment and observation compared, 332
Eyelids, anatomy of the . . 217

Factories, on the climate of . 386
Fauces, peculiar affection of . 565
Fever, on the causes of . . 270
influence of age and sex on . ib.
on sugar of lead in . . 239
intermittent, at Lisbon . 558
Final causes, Mr. Whewell on . 338
Fistula in ano . . . 481
Fletcher, Dr. his Physiology . 75, 92
Flourens on the Spinal Marrow . 532
Fœtus, amputation of its limbs . 614
Fork extracted from the body . 609
Fractures, on disunited . . 460
on the cure of . . 248

PAGE
Fracture of the ribs . . 574

Ganglionic nerves, influence of . 336
Geary, Dr. or Fever . . 270
Glands, intestinal, Dr. Albers on . 184
Glottis, œdema of . . . 431
spasm of . . . 438
Glisson, M. his doctrines of the spinal cord . . . . 523
Grainger, Mr. on the Spinal Cord . 486
his experiments on salamanders . . 514
Graves, Dr. on the treatment of cholera . . . . 280
Gusserow on Medico-legal Chemistry 212

Habit, influence of . . . 518
Hæmorrhoids, varieties of . . 482
Hall, Dr. on the Nervous System . 486
his discoveries . . 537
Hanging, Dr. Casper on . . 615
Headach, on aconite in . . 275
Health, various treatises on . 380
Heart, Dr. Wardrop on diseases of . 146
on the sounds of the . 425, 589
rupture of . . . 607
Hecker, Dr. on the Plague . . 191
Hernia, radical cure of . . 300
Hidrosis, case of . . . 290
Home, Dr. his Infirmary Report . 601
Obituary of . . 628
Homicide, Mr. Watson on . 171
Hunter, John, his treatise on Syphilis 230
on the Spinal Cord . 528
Hydrocele, acupuncture in . 279
Hydrocephalus, accidental case of . 565
Hygiène, public, Duchatelet on . 447
Hysteria, cases of . . . 601

Ice, treatment of aneurism by . 568
Inductive Sciences, history of . 317
Infirmary, Edinburgh, report from . 601
Glasgow, report from . 607
Influenza in Birmingham . . 214
Lisbon . . 557
Copenhagen . . 558
Insane, moral management of . 239
Insanity, Mr. Brown on . . 65
Insects, on the sensibility of . 545
Iodine, Dr. Cogswell on . . 162
Iris in birds, structure of . . 553

Johnson, Dr. on Health . 380, 393
Joints, on diseases of . . 461
Jones, Mr. on Staphyloma . . 612
Judd, Mr. on Syphilis . 2, 29

Kidney, Bright's disease of the . 554
Kramer, Dr. on diseases of the ear . 216

Laennec's Treatise, notes on . 423
Lallemand, M. on stricture . 572
Larynx, on diseases of . . 424

21

PAGE
Larynx, wounds of . . 465
Laryngitis chronic, treatise on . 424
acute . . 430
Laryngismus stridulus . . 595
Law, Mr. on Arsenic in Plethora . 605
Lawrence, Mr. on Ruptures . 547
Laycock, Mr. on Saliva . . 268
Laycock, Mr. on hysteria . . 601
Lead, acetate of, in fever . 239
- cholera . 280
Lee, Dr. on Supernumerary Mammæ . 585
Leeds, Philosophical Society of . 211
Legallois, on the Spinal Marrow . 530
Leitao, Dr. on Intermittents . 588
Life, Reil on . . . 78
Arnold on . . . 91
Hunter on . . 47
Light, its effects on health . 405
Lime, chloride of, in wounds . 575
Liston, Mr. his Practical Surgery . 457
Lithotrity, progress of . . 249
Lorinser, Dr. on the Plague . 191
Louis, M. on Fever . . 549
his numerical method . 154
Love, symptoms of . . 404

Mackintosh, Dr. Obituary of . 311
Magnetism, animal . . 598
Malcolmson, Mr. on Beriberi . 122
Mammæ, supernumerary . 385-594
Man, Dr. Prichard on . . 543
Marriage, Dr. Ryan's book on . 442
Mayo, Mr. his Physiology . 78-98
Mr. on Health and Digestion 380-388
on the spinal cord . 486-533
Medicine, Dr. Williams' Elements of 353
Dr. Billing's Principles of 200
Memoirs of the Bologna Society . 167
Parisian Society . 154
Menstruation, serotine . . 256
Milk and whey, effects of . . 565
Monsters, account of . 169-170
Morphia, endermic use of . 348
chemical detection of . 213
Morphology, Dr. Carus on . 85
Mothers, hints to, on health . 215
Müller on the Anatomy of the Nervous System . . . 293
Müller, on the Reflex Function . 536
his Manual of Physiology . 75
Munk, Dr. on Delirium Tremens . 600
Muscular motions, arrangement of . 505

Nagle, Dr. on the Stethoscope . 596
Nævus, fatal case of . . 613
Nerve, optic, origin of . . 593
Nerves, Dr. Reid's experiments on 8th pair of . . . 308
Dr. Berres on the structure of 219
cerebral, Mr. Grainger on . 498
Nervous System, Müller on the . 293
Dr. Verity on . 540

PAGE
Nervous System, in the lowest animals 489
Dr. Hall on . 486
its modifications . 493
Neuralgia, on the nature of . 276
Newport, Mr. his discoveries . 615
Nitrous oxide, curious effects of .114
Nose, operation for a new . 245
Numerical method, M. Louis on . 154
not always applicable 157

Oesterlen on Syphilis . . 1-28
Opium, its use in ulcers . . 197
Ophthalmia, calomel in . . 251
Oppenheim, Dr. on Syphilis . 2-29
Osborne, Dr. on Neuralgia . 276
Ovum, on the development of . 555
Owen, Mr. on Dr. Hall's discoveries 538

Pancreas, inflammation of the . 567
Paralysis, partial, of the face . 281
of the muscles of inspiration 133
Mr. Mayo on . . 107
Paris, Dr. on Diet . . 380-403
Pericarditis, Dr. Watson on . 604
Phthisis, laryngeal . . 424-433
on inhalation in . . 606
Physiologists, a genus irritabile . 620
Physiology, various treatises on . 75
an inductive science . 317
on the mode of studying 340
Plague, Hecker and Lorinser on . 191
Plague, Dr. Bulard on the . 560
Plethora, arsenic in . . 605
Pleura, fibrous membrane of . 220
Poisons, morbid, Dr. Williams on 353
Poor Law, medical relief under . 310
Porter, Mr. on the Larynx . 423
Poultices, objections to . . 464
Powel, Mr. on the Inductive Philosophy 548
Pregnancy, extra uterine . 254
auscultation in . 167
Prichard, Dr. on Man . . 543
Prochaska, on the Reflex Function . 623
Pulse of Infants . . 268
Pus, effect of, on bone . . 251
Pyrexia, Dr. Clutterbuck on . 185

Quain, Mr. his Anatomy . 216
Quina, used endermically . 350

Rabies in animals . . 262
Radford, Mr. on inversion of the uterus 288
Rectum, on diseases of the . 480
Reflex Function, Prochaska on . 622
Reid, Dr. on the 8th pair of nerves 308
Respiration, nature of movements in 511
Resuscitation after interment . 582
Revaccination in Prussia . . 210
Rheumatism, Dr. Zeroni on . 230
Ribs, fracture of, by contre-coup . 574
Riofrey, Dr. on Female Education 300-408
Robertson, Dr. on Diet . 380-397

PAGE
Ruptures, Mr. Lawrence on . 547
Ryan, Dr. his book on Marriage . 442
Ryland, Mr. on the Larynx . 424

Saliva, chemical reaction of . 268

Saunders, Mr. on the Teeth . 205

Scarlatina, Dr. Williams on . 363

Schlegel, Dr. on Suicide . 368

Shoulder joint, on dislocation of the 569

Scirrhus, fatal case of . . 419

Scudamore, Sir C. on Inhalation . 606

Shaw, Mr, on Facial Palsy . 281

Skey, his mode of treating ulcer . 195

Skin, medication by the . . 343

Small-pox, morbid anatomy of . 469

on internal parts . 476

history of, in Denmark . 207

Smith, Dr. his Philosophy of Health 380

Sodium, chloride of, in fever . 563

Solly, Mr. on the Optic Nerve . 593

Stains on linen, tests of . . 206

Spasmodic affections, treatment of . 275

Speculum uteri, new . . 255

Spinal cord, on the physiology of . 486

anatomy of . . 496

history of its physiology 523

affected in beriberi . 118

central softening of . 182

Dr. Smith on . . 224

Spinal Curvature, Mr. Mayo on . 334

Dr. Stromeyer on 133

Spinal nerves, on the roots of . 496

Stammering, new mode of treating . 568

Staphyloma, Mr. W. Jones on . 612

Statistics of diseases . . 262

Stethoscope, its use in pregnancy . 596

principles of the . 267

Stokes, Dr. on Diseases of the Chest 423

Stricture, new mode of treating . 572

Stromeyer, Dr. on Spinal Curvature 133

Strychnia, used endermically . 350

on the action of . 221

Sugar in dysentery . . 243

Suicide, treatises on . . 368

morbid appearances in . 373

statistics of . . 371

Surgery, Mr. Cocks's Operative . 544

Mr. Liston s Treatise on 457

Sydney, diseases at . . 261

Syphilis, various treatises on . 1

non-mercurial treatment of 3

natural history of . 7

treatment of, simple . 17

mercurial . 21

PAGE
Syme, Mr. on Diseases of the Rectum 480
Syringe, female, improved . 307

Teaspoon swallowed, case of . 252
Teeth, the, a test of age . . 205
Tendo Achillis, division of . 285
Tendons, on the physiology of divided 550
Testis, on the removal of the . 416
Tetanus, cases of . . . 226
Thrombus, fœtal, etiology of . 584
Ticknor, Dr. his Philosophy of Living 381
Tiedemann on the Negro Brain . 585
Trephine, on the use of . . 459
Treviranus, Obituary of . . 313
Tricocephalus dispar . . 594
Tumours, Dr. Warren on . 414
Turner, Dr. his Chemistry . 216
Typhus Fever, Dr. Williams on . 357

Ulcer, Mr. Skey on . . 195
Umbilical cord, injections into . 257
Urinary organs, diseases and injuries of 467
Urine, its state in beriberi . . 122
Urine blue . . . 266
Uterus, inversion of . . 288
case of ruptured . . 581

Vagitus uterinus, case of . . 257
Velpeau, M. on dislocation . 569
Venereal disease, treatises on . 1
Verity, Dr. on the Nervous System 540
Vitreous humour, ossification of . 253
Volition and emotion . . 503

Walcheren, diseases of the army in 290
Wardrop, Dr. on the Circulation . 146
Warming and Ventilating, Dr. Arnott on 546
Warren, Dr. on Tumours . 414
Watson, Dr. on the Aortal Valves . 600
Watson, Mr. on Homicide . 171
Watson, Dr. on Pericarditis . 604
Wendt on the Small Pox in Denmark 207
West, Dr. Obituary of . . 311
Whewell, Mr. on Inductive Science 317
Whipple, Mr. on dividing the tendo Achillis . . . 285
Whytt, Dr. on the Spinal Cord . 523
Williams, Dr. on Morbid Poisons . 353
Wounds, medico-legal history of . 172
Wounds, on the union of . . 458
painful, remedy in . 575
incised, new mode of treating 576
treated by cold water . 574

END OF VOL. V.

Printed by C. Adlard, Bartholomew Close.

